# Twelve Years of Postgraduate Palliative Medicine Training in Finland: How International Guidelines Are Implemented

**DOI:** 10.1089/pmr.2021.0020

**Published:** 2021-09-17

**Authors:** Aija Vanhanen, Leila Niemi-Murola, Reino Pöyhiä

**Affiliations:** ^1^ Helsinki City Laakso Hospital, Helsinki, Finland.; ^2^ Department of Anesthesiology and Intensive Care Medicine, University of Helsinki, Helsinki, Finland.; ^3^ Department of Oncology, University of Helsinki, Helsinki, Finland.; ^4^ The Palliative Care Center, South Savo Social and Health Care Authority (Essote), Helsinki, Finland.

**Keywords:** curriculum, evaluation, medical education research, palliative care

## Abstract

***Background and Objective:*** The European Association for Palliative Care (EAPC) published recommendations for postgraduate education in palliative medicine in 2009. However, it is currently unknown how the EAPC remommendations are implemented in national programs, as audits of them are lacking. In Finland, the national society of palliative medicine has been organizing postgraduate palliative medicine training for experienced physicians since 2008, but the program has not been audited. The aim of this study was to perform a comprehensive analysis of the program.

***Design:*** In 2018–2019, a questionnaire on the Finnish Training Program for Palliative Medicine Competence was sent to past participants and delivered in person to current trainees. Learning outcomes were assessed with validated instruments for received skills and attitudes. All available educational archives were examined as well.

***Results:*** Forty-five (32 %) out of 155 specialists and 13 (38 %) out of 34 trainees responded. According to their assessments, the training provided them well with most skills required to work as palliative care specialists, but poorly with research capabilities. However, the Finnish program covers the EAPC guidelines well. Problem-based education, group work, and clinical excursions have been added to the latest curriculum. Maturation through work is needed for administrative and consultant competences.

***Conclusion:*** The EAPC guidelines can be included in a national course. The course had an important positive influence on the attitudes and learning of physicians in palliative medicine. The development of the education would benefit from pedagogical consultation. Uniform standards for auditing national programs should be developed.

## Introduction

In 2009, the European Association for Palliative Care (EAPC) published recommendations for the development of postgraduate curricula leading to certification in palliative medicine,^1^ which still is the golden standard for specialist training in Europe. Even so, there is no information available on how these recommendations are implemented in national programs. A recent international web survey,^2^ based on specialists' views, outlined current needs and structure for postgraduate education and proposed creating a pan-European program for postgraduate training in palliative medicine. However, the possibility of an international curriculum has also been questioned, due to the countries' different cultural and educational structures.^3^

Currently, palliative medicine is a recognized specialty only in a few European countries; Denmark, Ireland, Malta, Poland, Slovakia, and the United Kingdom; and a subspecialty or special competence in many others.^4^ Specialist training in palliative care and medicine is organized differently in different countries. A study found that 18 of the 35 European countries had formal programs in palliative medicine, organized in various ways, including those provided by universities, national palliative care societies, and postgraduate courses. However, very little detailed information is available on how these programs are executed. In fact, a recent study aimed at executing a meta-analysis on the contents of these educational programs, but it found only six to analyze.^5^ So far, the evaluation of palliative care curricula has been based either on web-based questionnaires^1,3^ or on expert panels.^3,6–8^ Unfortunately, there seems to be a lack of published feedback from specialists, who would be experts in assessing the curricula they have attended.

In Finland, postgraduate education in palliative medicine has been available for experienced physicians since 2008. This two-year program includes teaching in key topics of palliative care as well as practical training. Although the training started 12 years ago, the effect of this program has not been evaluated or audited. The aim of this study was to perform a comprehensive analysis of Finnish postgraduate education in palliative care. A special interest was to assess the outcome of the program and compare it with the EAPC standards.

## Methods

**FIG. 1.**
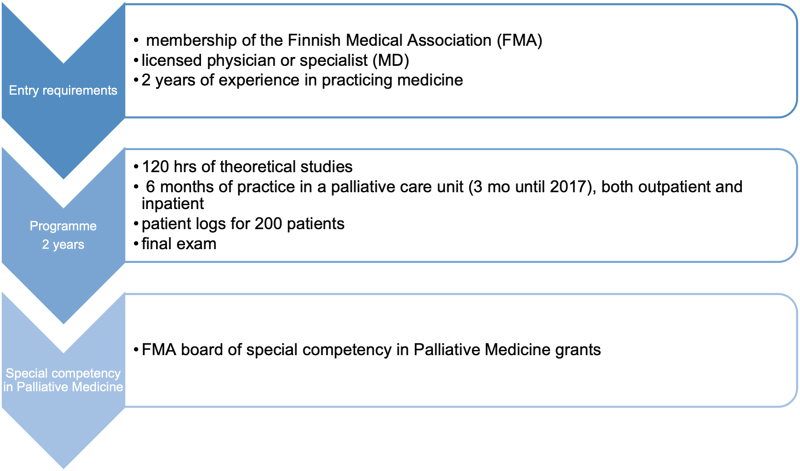
Path to special competency of Palliative Medicine for medical doctors in Finland.

The study was approved by the University of Helsinki Ethical review board in humanities and social and behavioral sciences on March 24, 2017 (record number 54/2017). The Finnish program for postgraduate education in palliative medicine is organized and supervised by the Finnish Society for Palliative Medicine (FSPM). After the applicant has completed this two-year program, the Finnish Medical Association (FMA) certifies them with special expertise (Fig. 1).

Documents covering palliative care special units, the planning and execution of training courses, course programs, participants' applications, and board meetings regarding the courses were obtained from the FSPM electronic archives and examined. The contents of the first (2008) and last theoretical courses (2019) were compared with the EAPC curriculum. The exact hours of the daily programs were calculated and transformed into teaching periods of 45 minutes, which is considered one teaching period in the special competence board. Detailed information about questions and exam grades and numbers of awarded qualifications was received from the FMA. Additional information was gathered from personal communications with individuals responsible for education at the FSPM and FMA. The questions and passing grades of the exams were analyzed.

In 2018, a questionnaire survey about the specialist competence program in Finland was sent to all physicians with special competence in palliative medicine and was delivered personally to trainees on the last day of their course. The questionnaire was re-sent five months after the first delivery. The questionnaire consisted of demographic and education sections. Information about the respondents' age, gender, specialty, and workplace was collected by using both open-ended and predefined questions. A Finnish translation of the EAPC recommendations on training for postgraduate palliative medicine was included in the education section. The doctors were asked to evaluate, using a 10-point numeric rating scale, how the Finnish program corresponded with the EAPC recommendations in both quantity (0 = no teaching at all, 10 = sufficient amount of teaching) and quality (0 = bad, 10 = excellent). In addition, the respondents were asked to evaluate the teaching methods used. Finally, using the EAPC curriculum outcome and role model definitions, the respondents were asked to evaluate how well the program had provided them with the competences and desired skills needed as a palliative care specialist by using a numeric Likert scale of 1–7 (1 = does not apply to me, 7 = fits well to me).

### Data analysis

The responses were analyzed after being anonymized. The distribution of the metric data was assessed by using a *z* test for normality, with *z* < 3.29 indicating normality.^9^ The data are presented in means or percentages, with 95% levels of confidence or standard deviations (SDs). Pair-wise comparisons between rankings by specialists and trainees were made by using confidence levels, with *p* < 0.05 indicating significant difference.

## Results

Ninety documents on palliative training courses from the electronic archives of the FSPM were analyzed, whereas five specialists from the FSPM and FMA were interviewed. Forty-nine (32%) of the 155 specialists and 13 (38%) of the 34 trainees responded to the questionnaire. The demographics of the respondents are given in Table 1. The responding specialists consisted of 13 geriatricians, 12 oncologists, 11 general medicine specialists, 5 anesthesiologists, 1 pulmonologist, 1 pediatrician, 1 clinical pharmacologist, and 5 physicians without specialties; whereas the trainees were 5 geriatricians, 3 general and 2 internal medicine specialists, 1 oncologist, 1 anesthesiologist, and 1 pulmonologist.

**Table 1.** Respondents' Demographics

	Specialists	Trainees
Responses	49	13
Female, *n* (%)	39 (80)	11 (85)
Male, *n* (%)	10 (20)	2 (15)
Age in years, median (range)	54 (34–70)	44 (32–55)
Age distribution, *n* (%)		
<25	0 (0)	0 (0)
25–34	1 (2)	2 (15)
35–44	5 (10)	2 (15)
45–54	19 (39)	8 (62)
55–64	16 (33)	1 (8)
65–69	6 (12)	0 (0)
≥70	1 (2)	0 (0)
Unknown age	1 (2)	0 (0)
Current workplace, *n* (%)		
Local hospital	9 (18)	1 (8)
Home care	1 (2)	4 (31)
Hospital at home	6 (12)	2 (15)
Central or university hospital	16 (33)	5 (38)
Private clinic	2 (4)	0 (0)
End of life care	5 (10)	0 (0)
Other or N/A	10 (20%)	1 (8%)

N/A, no answer.

### Educational structure and participants

The program consists of 150 hours of theory, and two years of full-time clinical work in a palliative care service, of which a minimum of six months is to take place in a special palliative unit. Details of the program are presented in Figure 1. Each trainee has a personal tutor from the FSPM.

The prerequisites for the participants are: a specialist or an experienced physician with more than two years' work history and a demonstrated interest and experience in palliative care. They should also come from different parts of Finland. The participants are chosen by the FSPM.

The number of applicants has increased over the years: Although all 30 applicants were accepted into the first courses beginning in 2008 and 2010, between 2014 and 2020 a total of 142 physicians (66%) out of 215 applicants were accepted onto the course.

### Special palliative units

There are currently 30 special palliative care units approved by the FSPM to provide full-time internships for palliative care specialist training in Finland. The requisites for such units are: at least one physician with special competence in palliative medicine and in supervision (tutorship); most of the patients in the unit must be palliative and/or in end-of-life care for 24/7; a multi-professional palliative care team with a physician, a nurse, and a special worker (for example, social worker, clergy, physiotherapist); and the unit has an outpatient service (outpatient clinic, hospital-at-home, home care) as well as an inpatient ward. In addition, the participants' full-time work must be only in palliative care and a palliative care specialist must be available for supervision every day. The special board of palliative medicine at the FMA approves the applications for a special unit twice a year and audits the units once a year.

### Content of the theoretical course

The Finnish training course includes 120 hours of theoretical education, which is delivered in several 2- to 3-day sessions over 1.5 years. The cost of the education is 1800 €, which often is paid for by the participants' employers. The participants must gather 30 hours of additional education in national and international courses on palliative care/medicine. Detailed documented information about the Finnish programs has been available since the first course. The training days have been held at different universities around the country to facilitate geographical accessibility. The content and volume of the courses has changed significantly over their 10 years (Table 2). More problem-based education, group work, and excursions to palliative care units have been included in the latest curriculum. The amount on pain medicine and on AIDS-related issues has diminished in the newer program, whereas education about advanced care planning, palliative sedation, and the roles of radiology and physiotherapy has increased. Teaching methods are mostly lectures, group work, case studies, and seminars. Significant changes in the methods of teaching were observed in role-playing exercises, which were used very little in the last course.

**Table 2.** Contents of the Finnish Curriculae in 2008–2009 and 2018–2019, Following the Main Categories Laid Out by the European Association for Palliative Care

Subjects (EAPC heading)	2008–2009	2018–2019
1. Introduction to palliative care (L)		
History, philosophy, and definitions	1	1
2. Physical care and treatment (L)		
Management of life-limiting, progressive diseases		
Advanced care planning	0	2
Specific diseases processes		
AIDS	2	0
Cancer	13	13
Cardiovascular and pulmonary diseases	2	3
Dementia, geriatric patients	8	4
Developmental disabilities, congenital disorders	1	1
Infections	0	1
Liver diseases	0	1
Neurological diseases (ALS, poststroke syndrome)	1	3
Renal diseases	1	1
General principles of symptom management		
Epidemiology of symptoms	2	1
Anorexia, cachexia, and fatigue	2	2
Dermatological problems	0	1
Dyspnea and other respiratory symptoms	2	1
GI-tract symptoms and nutritional issues	3	3
Gynecological problems	0	1
Delirium and other psychiatric symptoms	0	2
Pain	17	4
Urological problems	0	1
Palliative sedation	0	1
Complimentary treatments	2	1
End-of-life care in ICU	0	1
Radiological issues and procedures	0	1
Nursing issues	1	1
3. Psychosocial Care and interventions (L, D)		
Communication	10	11
4. Culture, Language, Religious and Spiritual Issues (L)		
Existential issues	0	3
Cultural issues	3	2
5. Ethics (L)	3	2
6. Legal (L)	0	0
7. Team work (L, D)	5	5
8. Teaching and learning (L)	1	2
9. Research (L)	0	1
10. Management		
Organization of palliative care services (L, D)	2	5
Human resources	2	3
Excursions to palliative care units	3	4
Group work on daily topics (G)	9	11
Problem-based learning and independent study (D)	10	9
Total	120	120

The numbers refer to teaching hours of 45 minutes each. The amount of problem-based learning and independent study is an estimation.

ALS, amyotrophic lateral sclerosis; D, discussions; EAPC, European Association for Palliative Care; G, group discussion; GI, gastrointestinal tract; ICU, intensive care unit; L, lecture.

All teachers of the courses were experienced physicians and other practitioners with either vast knowledge or special competence in palliative medicine. The number of teachers per course increased from 47 to 61. Only seven teachers remained the same in all courses.

### Examinations

A written exam of six hours has been included in all courses, excluding the very first one in 2008. The trainees could take the exam at any point of the course. The exams were organized twice a year if needed. A total of 17 exams were organized in 2011–2020. The questions were prepared and answers graded by members of the specialist board of the FMA, which was responsible for standardization. The exam had a general part and an additional part tailored for the specialty in the form of essays and clinical cases. Multiple-choice questions were not used. Two typical questions are: (1) Differences between opioids in medical use and opioid rotation; and (2) Treating the delirium of a dying patient. Similar topics were also included in the additional questions tailored for the specialty. These questions have remained rather similar over the years. All questions could be answered, with knowledge provided during the training. The passing score was 50% of the maximum points. If a trainee gets <20 out of 36 points, two examiners must agree on giving a passing grade. All participants have passed the exam with an average grade of 27.3 (SD 2.97) out of 36.

### Comparison of the national and EAPC course

The evaluation given by specialists and trainees about how the national course covered the main categories of EAPC guidelines is given in Table 3. Overall, the quality was ranked higher than the quantity. Compared with the EAPC curriculum, the Finnish trainees felt that administrative issues (5–6/10), research in palliative care (6–7/10), teaching materials (7/10), the role of the hospital pharmacy (7/10), care of deaf and violent patients (7/10), and treatment of hypoglycemia (8/10), acute dystonia (8/10), and serotonergic crisis (7.5/10) were inadequately covered in the training.

**Table 3.** European Association for Palliative Care Coverage

EAPC main category	Specialists			Trainees		
	Quality	Quantity	*N*	Quality	Quantity	*N*
Introduction to palliative care	8.2 (0.6)	7.6 (1.0)	17	7.2 (1.2)	6.9 (1.3)	11
Physical care and treatment	8.1 (0.8)	7.9 (0.9)	15	6.8 (1.4)	6.1 (1.7)	11
Psychosocial care and interventions	7.9 (0.8)	7.4 (0.9)	13	7.5 (1.2)	6.9 (1.7)	10
Culture, language, religious and spiritual issues	8.5 (0.6)	8.3 (0.6)	12	8.0 (1.0)	7.9 (1.3)	10
Ethics	8.6 (0.7)	8.4 (0.8)	12	7.2 (1.2)	6.6 (2.0)	10
Legal frameworks	8.1 (1.0)	7.0 (2.7)	12	6.8 (1.6)	5.2 (2.3)	9
Teamwork	8.1 (0.7)	8.0 (1.4)	13	7.6 (1.1)	6.7 (1.9)	10
Teaching and learning	7.4 (1.3)	6.5 (2.8)	12	6.2 (2.8)	4.5 (2.6)	9
Research	6.5 (0.9)	1.7 (3.4)	11	5.9 (2.5)	3.6 (2.3)	8
Management of palliative care	6.9 (1.4)	5.2 (3.4)	13	4.8 (2.6)	3.8 (2.4)	7

The specialists' and trainees' mean rankings of the main items in the Finnish curriculum, compared with the EAPC curriculum, with 95% intervals of confidence using the main thematic categories of EAPC curriculum. Both the quality and the quantity of teaching were assessed. 0 = item was not included, or the quality of its teaching was poor, 10 = fully included, or the teaching was excellent. Statistical differences between specialists and trainees were observed. *N* = number of responders.

### Outcomes

Both the specialists and trainees estimated that the course had provided them with most skills required to work as a palliative care specialist; but on the other hand, with poor capabilities to research palliative medicine (Table 4). There were differences in the self-evaluation between the specialists and trainees (Table 5) regarding their maturity as palliative care specialists in their roles as a consultant and manager.

**Table 4.** Received Skills

Item	Specialists	Trainees
1. Palliative medicine specialist	6.2 (0.3)	5.9 (0.3)
2. Interactive skills	5.9 (0.5)	5.6 (0.5)
3. Collaboration	5.6 (0.4)	5.8 (0.8)
4. Leadership in palliative care	4.7 (0.6)	5 (0.7)
5. Opinion leader in society	4.5 (0.8)	4.8 (0.7)
6. Teaching skills	4.8 (0.7)	4.5 (0.5)
7. Research	2.7 (0.8)	2.9 (0.8)

Specialists' and trainees' assessments of the course providing them with the essential skills to carry out different roles in as a palliative are professionals, as defined in the EAPC curriculum. Mean values and 95% intervals of confidence are given. (0 = I did not gain any skills, 7 = I gained excellent skills during the course).

**Table 5.** Self-Reflection of the Specialists (*N* = 42) and Trainees (*N* = 13) in Palliative Medicine after Passing the Training Course

Item	Specialists	Trainees
1. I have expert knowledge of pathophysiology, symptom management, psychosocial and spiritual issues related to life limiting illness and imminent death	6.1^*^ (0.2)	5.2 (0.5)^*^
2. I understand the experience of disease from the perspective of the patient and the meaning and consequences of illness to the patient and their family	6.1 (0.2)	5.5 (0.4)
3. I can make appropriate clinical decisions to provide medical care that is structured around the patients' and families' needs, their understanding and priorities, with the aim of maximizing quality of life, relieving suffering, supporting the family, and normalizing their experiences	6.8 (0.1)	6.5 (0.3)
4. I have particular expertise in the management of patients within the home, as well as the hospital and hospice	6.2 (0.3)	5.5 (0.5)
5. I understand the natural history and role of disease-specific treatments in the management of advanced cancer and other progressive life-limiting illnesses	6.2 (0.3)	5.7 (0.4)
6. I practice culturally responsible medicine with understanding of the personal, historical, legal, cultural, and social influences on patients and families	6.5 (0.2)	5.9 (0.5)
7. I am able to provide expert advice as a consultant	6.4 (0.2)^*^	5.7 (0.3)^*^
8. I establish therapeutic and supportive relationships with patients and their families based on understanding, trust, empathy, and confidentiality, understanding that this relationship may cover the time of bereavement	6.6 (0.2)	6.1 (0.4)
9. I am an expert in discussing end-of-life issues with patients and their families as well as with other medical specialists	6.3 (0.3)	5.6 (0.5)
10. I am able to act as a palliative care expert in euthanasia discussion and know the current Finnish laws about euthanasia and ethical framework in my country	5.7 (0.4)^*^	4.1 (0.7)^*^
11. I am able to sensitively explore the patients' concerns across physical, psychological, social, cultural, and spiritual domains	6.0 (0.3)	5.1 (0.5)
12. I can communicate effectively with patients, their families, and other health professionals involved in the patients' care	6.5 (0.2)	5.6 (0.6)
13. I can manage my own time and resources effectively	5.3 (0.4)	4.5 (0.8)
14. I participate regularly in further education in palliative medicine	5.8 (0.5)	5.8 (0.6)
15. I can manage administrative managerial tasks in palliative care, pertaining to finances, human resources, quality oversight, and information management	4.8 (0.5)^*^	2.9 (0.7)^*^
16. I am able to effectively allocate health care resources in the care of my patients	5.7 (0.3)	5 (0.7)
17. I can alleviate the physical symptoms of my patients in different stages of their diseases	6.3 (0.2)	5.8 (0.4)
18. I can meet the social needs of my patients	5.9 (0.2)	5.5 (0.5)
19. I can meet the spiritual needs of my patients	5.7 (0.3)	5.1 (0.6)
20. I practice self-reflection continuously and am involved with professional development	6.1 (0.3)	5.5 (0.7)

Mean values with 95% intervals of confidence are given.

Statistical differences between specialists and trainees are marked with ^*^p < 0.05.

0, not applicable; 7, applies to me perfectly.

## Discussion

In Finland, two years of postgraduate training in palliative medicine is provided by the FSPM. After the training, the FMA qualifies the doctor with a special competence in palliative medicine. In the current study, Finnish physicians evaluated that the Finnish curriculum covers most of the content of the EAPC curriculum well. Most of the training deals with palliative oncology and the dying process, but administrative issues, research, and spiritual support are covered less. Additional education tailored for different specialties must be obtained by the specialist physicians themselves. The educational content of the Finnish courses has changed during the observational period of 12 years along with changes in medical care. According to their self-evaluation, all the trained doctors reported that they had received sufficient skills to work as a palliative consultant in all but academic capabilities. However, there is a lack of multidisciplinary education and an obvious need to modernize the pedagogic methods of the teaching.

We have provided an audit of a national palliative medicine postgraduate program. The current audit was based on (1) comprehensive examination of teaching programs and exams, (2) analysis of documentation in the pedagogic archives of the education provider and personal interviews, (3) evaluations by previous and currently attending students, (4) comparison of the national and EAPC curricula, and finally (5) assessment of the learning outcomes. In this study, for the first time, not only experts but also physicians who had attended the training compared the experienced education with the European standard.

Both developing and maintaining common international or pan-European training programs, which have been suggested,^2^ would benefit from a detailed systematic analysis and auditing of current national programs. Postgraduate training programs are still very differently organized around the world. Centeno et al.^3^ reported that the length of curricula varied from 100 hours to 2 years in different European countries. In Finland, the expected time required for the competence is 2 years and the volume of theoretical teaching 150 hours. We found that the national course provided only 120 hours and that the rest must be covered through supplemental education, tailored for individual needs. Obviously more important than the volume of teaching would be the defined competences,^10^ which were most recently suggested by Paal et al.^2^ Concomitantly with commonly accepted competences,^2,11^ and goals, a uniform method of auditing the educational programs should be developed in EAPC. The method of the present assessment is highly supported in a recent paper by Moreau.^12^

Our observations show that the Finnish palliative medicine specialist training program covers most parts of the EAPC program. The Finnish specialists and trainees gave rather high overall rankings to the quality and quantity of the program. The trainees were more critical than the specialists, which can be explained in part by their fresh memory. The lowest rankings were given to teaching on administrative issues and research, which are not very well covered in the Finnish curriculum. Emmerich,^13^ among others, points out the importance of administrative education in palliative care. However, the managerial and administrative skills are rather generic and can be learned on other courses, which leaves more time for substance-specific education in the palliative medicine training program.

The largest amount of the Finnish curriculum is dedicated to the palliative care of cancer patients. This is evident in both the number of teaching hours and the exams, where almost all patient cases dealt with cancer-related palliative care problems. This may be a matter of concern, since still too many patients with advanced non-malignant diseases do not receive adequate palliative care.^14^ However, a positive observation in this study was that the exams aligned with the teaching.

The current analysis also observed several important changes in the Finnish curriculum over time. There are many reasons for this. First, the group in charge of the course and the teachers have changed. Only seven teachers have been the same across all courses. Second, the amount of group teaching has increased, although other didactic methods have remained rather similar. Third, teaching about AIDS has diminished as advanced care planning, which was not widely understood in Finnish clinical practice 12 years ago, has increased. The incidence of HIV infection has remained very low in Finland, but there has been a lack of formal education about advanced or end-of-life care planning in incurable diseases. Finally, pain-related issues have diminished in the curricula. Interestingly enough, a similar observation was made in a recent systematic analysis by Turrillas et al.^5^ In Finland, another two-year program for special competence in pain medicine is available, which may be the reason for the diminished pain-related teaching in the palliative medicine postgraduate curriculum. However, assessment of the attitudes and the self-evaluation showed that the curriculum positively influenced the personal development of the participants. Naturally, maturity in the profession comes through experience: This study shows that the specialists were more confident to act as consultants and managers than the trainees.

We believe that the current curriculum could be improved. Modern, activating didactic methods such as learning diaries, simulations, or distance learning have also been proven cost-effective methods in palliative care education.^15^ In addition, positive experiences have been gained in interprofessional teaching,^16,17^ which is so far lacking in the Finnish curriculum. We agree with Paal et al.,^2^ who propose even more methods: The students would most likely benefit from blended, interactive learning and storytelling by real patients or carers.

An important point of criticism concerns the organization of the program. The national course is organized and hosted by a special group of doctors in the FSPM. The scientific qualifications of the teachers and the content of the program are monitored by the FMA. This study has shown the importance of faculty development in the construction of our educational program. We think that postgraduate education in palliative medicine should be provided at universities, not only in the relevant societies. So far, to receive certification, an applicant must also be a member of the FMA, the physicians' labor union, but this should not be a requirement. A careful analysis of the program, definition of learning outcomes, and choosing evidence-based educational methods are important in developing teaching in faculties as well.^17,18^ All the educational curricula should be regularly audited and qualified.

In Finland, the current number (192) of physicians with special competence in palliative medicine is 3.4/100,000 inhabitants, which is more than three times the average in Europe (1/100,000) and closer to that in the United States (1.9/100,000).^19^ However, we estimate that the number of palliative medicine specialists does not meet the needs of palliative care in the future. The lack of appropriate education and knowledge and shortage of qualified doctors is also a matter of concern in other countries.^2^ A recent study proposed that two to three times more specialists are needed in Europe in home-based palliative care alone in the future.^20^ In the United Kingdom, Etkind et al.^21^ estimate an increase of 24%–47% of palliative care services by 2040 simply because of aging. This would, undoubtedly, mean even greater needs in palliative medicine specialists.

### Limitations

One limitation of this study is the relatively low response rate (30%). Another hindrance is that the internships in palliative units could not be evaluated retrospectively. However, 45 physicians who had all received the training filled in the questionnaires. In addition, their specialties well represent those of all specialists with palliative competence. Moreover, the distribution of the rankings was normal with low intervals of confidence. Thus, we believe that the results of the pros and cons of the curriculum are reliable. Clearly, one merit of this study is that the assessment of the palliative medicine postgraduate program was done by the participants, and not based only on expert opinion.

## Conclusions

In conclusion, we have shown that the structure of the Finnish postgraduate palliative medicine training reflects well the current guidelines of the EAPC. The amount of cancer-related palliative care teaching has remained very high, but that of pain-related issues has declined. Although the theoretical education is based largely on lecturing and newer didactic methods are not used, the course has an important positive outcome in the attitudes and learned skills of physicians in palliative medicine. The future development of the education would undoubtedly benefit from pedagogic consultation. However, we propose that the postgraduate training program should be available in the Finnish medical faculties. The development of a pan-European program should include a uniform audit of currently existing national programs based on internationally agreed guidelines.

## Abbreviations Used

EAPC: European Association for Palliative Care
FMA: Finnish Medical Association
FSPM: Finnish Society for Palliative Medicine
SDs: standard deviations
